# Data-driven subtyping of Parkinson’s disease using MRI: current insights, challenges, and future directions

**DOI:** 10.3389/fnagi.2026.1819248

**Published:** 2026-09-03

**Authors:** Anupa A. Vijayakumari, Ken E. Sakaie, Hubert H. Fernandez, Benjamin L. Walter

**Affiliations:** 1 Center for Neurological Restoration, Neurological Institute, Cleveland Clinic, Cleveland, OH, United States; 2 Department of Diagnostic Radiology, Diagnostics Institute, Mellen Center, Cleveland Clinic, Cleveland, OH, United States

**Keywords:** clustering, data-driven subtyping, disease heterogeneity, machine learning, MRI, MRI-based subtypes, MRI feature-based subtyping, Parkinson’s disease

## Abstract

Parkinson’s disease (PD) is a heterogeneous neurodegenerative disorder marked by diverse motor and non-motor symptom profiles. Traditional symptom-based subtyping shows limited stability and lacks clear biological grounding. Integrating magnetic resonance imaging (MRI) with machine learning (ML) offers a promising avenue for defining biologically informed PD subtypes. This narrative review synthesizes evidence from MRI-based subtyping studies that used structural (T1-weighted), diffusion, functional, or multimodal MRI features as primary inputs for unsupervised or hybrid ML approaches to derive PD subtypes and outlines key methodological challenges and future translational needs. T1-weighted MRI studies consistently identify two to three subtypes characterized by distinct patterns of cortical and subcortical atrophy associated with variation in motor and non-motor symptoms. Although fewer in number, diffusion MRI studies have identified microstructural heterogeneity in PD. However, the findings remain heterogeneous and preliminary, and a stable subtyping framework has yet to be established. Multimodal MRI approaches show that combining modalities provides complementary insights into the neurobiology underlying PD heterogeneity but require further validation. Collectively, MRI-based subtyping shows promise for mapping clinical variability onto neuroanatomical patterns. At present, these subtypes are best viewed as research constructs that illuminate disease variability rather than clinical diagnostic tools. Translation into clinical practice will require addressing critical methodological gaps to achieve the reproducibility and prognostic utility necessary for precision medicine.

## 1 Introduction

Parkinson’s disease (PD) is a chronic, progressive neurodegenerative disorder that affects over 10 million people worldwide. In the United States alone, its prevalence is projected to exceed 1.6 million cases by 2037, with an estimated economic burden of $79 billion (Yang et al., 2020). The cardinal motor symptoms include bradykinesia, resting tremor, rigidity, and postural instability/gait difficulty (PIGD). Non-motor symptoms are equally prominent and encompass cognitive decline, autonomic dysfunction, rapid eye movement [REM] sleep behavior disorder (RBD), neuropsychiatric disorders, and sensory alterations (Chaudhuri et al., 2006; Kalia and Lang, 2015). Importantly, the expression and severity of these symptoms can vary widely among patients: for instance, one person may present with bradykinesia and tremor but minimal gait involvement or cognitive change, whereas another with a PIGD phenotype may already show cognitive and neuropsychiatric symptoms. This clinical heterogeneity has led to the view that PD is not a single, uniform disorder but a constellation of subtypes with overlapping symptoms (Greenland et al., 2019; Wüllner et al., 2023).

Traditional subtyping has relied on symptom-based classifications, such as the tremor-dominant (TD), akinetic-rigid (AR), and PIGD phenotypes. Among these, the TD subtype generally shows slower progression compared to the AR or PIGD subtypes (Jankovic et al., 1990; Kang et al., 2005). Classifications have expanded to include non-motor features and hybrid models combining motor and non-motor domains (Marras and Chaudhuri, 2016; Sauerbier et al., 2016; Rodriguez-Sanchez et al., 2021; Velucci et al., 2025). However, these symptom-based subtypes often shift over time as the disease progresses and are sensitive to factors such as medication effects, rater variability, and diagnostic drift, limiting their reproducibility and prognostic utility (Simuni et al., 2016; Mestre et al., 2018; von Coelln et al., 2021; Liotta et al., 2023). Attempts to replicate them across independent cohorts have been largely inconsistent (Mestre et al., 2018; Qian and Huang, 2019), highlighting the need for biologically grounded models that better reflect underlying disease mechanisms.

Biologically oriented models, particularly the brain-first versus body-first hypothesis and the α-synuclein origin and connectome model, propose that heterogeneity in PD stems from *where* α-synuclein pathology originates and *how* it propagates through the nervous system (Borghammer and Van Den Berge, 2019; Horsager et al., 2020; Borghammer, 2021). In the brain-first pathway, pathology begins in the olfactory bulb or amygdala and spreads caudally to the substantia nigra, typically resulting in asymmetric nigrostriatal damage and delayed autonomic symptoms. In the body-first pattern, pathology is proposed to originate in the enteric or peripheral autonomic nervous system and ascend via vagal and sympathetic routes, aligning clinically with prodromal RBD, autonomic dysfunction, and more symmetric striatal involvement. If entry site and propagation route differ across patients, then at least some of this heterogeneity should be visible as distinct patterns of atrophy, microstructural change, or network reorganization in the brain.

MRI can noninvasively capture structural, microstructural, and functional changes in the brain – including cortical and subcortical atrophy, white matter disruption, and connectivity alterations – providing more insight into underlying pathophysiological processes (Cao et al., 2024; Qi et al., 2025; Schröter et al., 2025; Tae et al., 2025). MRI is also widely available, does not involve ionizing radiation, and is well suited for repeated longitudinal assessments. However, it is important to distinguish the role of imaging in PD diagnosis from its role in biological subtyping. Molecular imaging techniques can provide more direct measures of specific pathological processes, such as presynaptic dopaminergic dysfunction, and are therefore often more closely aligned with diagnostic and target-specific biomarker applications (Nicastro et al., 2023; Yang et al., 2025). In contrast, conventional MRI is neither required for the clinical diagnosis of PD nor currently sufficiently sensitive or specific to establish the diagnosis at the individual-patient level.

The value of MRI for subtyping lies in its ability to characterize the spatial distribution and extent of brain abnormalities across clinically heterogeneous patients. Importantly, MRI provides whole-brain coverage and can capture both nigral and extranigral abnormalities, enabling the assessment of widespread disease-related alterations beyond the dopaminergic system. Structural, diffusion, susceptibility-sensitive, and functional MRI offer complementary information on brain morphology, tissue microstructure, iron accumulation, and network organization (Zarkali et al., 2024; Schröter et al., 2025). When these imaging features are analyzed using data-driven machine learning (ML) methods, they can reveal latent groups of patients who share neuroanatomical or connectivity signatures. Such imaging-derived subtypes are attractive because they are closer to putative disease mechanisms than purely symptom-based subtypes and can be mapped back to motor or non-motor phenotypes. In fact, several studies have reported MRI-based PD subtypes with distinct clinical associations and progression profiles (Uribe et al., 2019; Wang L. et al., 2020; Inguanzo et al., 2021; Cao et al., 2022; Vijayakumari et al., 2025a,b). However, a stable and widely accepted taxonomy has yet to emerge, likely due to methodological differences in imaging modalities, preprocessing pipelines, clustering strategies, limited external validation, and patient cohorts.

In this narrative review, we focus on MRI-driven subtyping in PD, i.e., studies that derive patient subtypes directly from structural, diffusion, functional, or explicitly MRI-based multimodal feature sets using ML approaches. We exclude studies that did not use MRI or that included MRI only as a minor component of a broader multimodal analysis without assessing its standalone contribution. By narrowing the scope in this way, we aim to isolate the unique contribution of MRI to biologically grounded PD subtyping. We first outline the general framework of MRI-based subtyping, including commonly used ML algorithms in this field and key methodological considerations. We then review studies that have applied these approaches to PD cohorts, and we conclude by highlighting current limitations and future directions needed to improve robustness, reproducibility, and clinical relevance.

## 2 Scope of the review

Eligible studies were original research articles in PD that (i) used MRI-derived features (structural T1-weighted, diffusion, and/or multimodal MRI), (ii) applied data-driven ML approaches to derive subtypes, (iii) reported clinical, cognitive, or longitudinal differences between the resulting subtypes (*post hoc*), and (iv) were published as full-length articles in peer-reviewed English-language journals. Studies were identified by searching PubMed, Google Scholar, and Web of Science using combinations of the terms “Parkinson’s,” “Parkinson,” “MRI,” “subtype,” “clustering,” “neuroimaging,” “data-driven,” and “machine learning,” prioritizing papers from the past decade (2016–2026). We excluded studies that (i) clustered patients purely on clinical (motor or non-motor) variables without MRI input or (ii) clustered patients on mixed clinical and multimodal imaging datasets (e.g., MRI plus molecular imaging such as positron emission tomography). This process yielded 10 studies for T1-based subtyping, 3 studies for diffusion MRI-based subtyping, and 3 studies for multimodal approaches.

## 3 Conceptual and methodological framework for MRI-based subtyping in Parkinson’s disease

The central idea of PD subtyping is that clinically heterogeneous patients can be stratified into latent subtypes (i.e., more homogeneous groups) that may reflect shared pathophysiological mechanisms. MRI-based subtyping aims to uncover this latent structure by analyzing high-dimensional imaging data. A common strategy is to use unsupervised machine learning, typically clustering, to group individuals with similar imaging profiles without predefined labels, making it well suited for exploratory analyses (Wang Y. et al., 2020; Eckhardt et al., 2023). However, the pronounced heterogeneity of PD has also led to more integrative frameworks that go beyond pure clustering. These hybrid methods combine unsupervised subtyping with supervised learning (i.e., classification guided by known clinical labels) or with disease-progression modeling, which estimates the temporal evolution of brain changes across subtypes. Table 1 summarizes representative ML algorithms that have been applied to MRI-based PD subtyping to date, including unsupervised, semi-supervised, and hybrid approaches.

**TABLE 1:** Machine learning methods used for MRI-based subtyping in Parkinson’s disease.

Method	Algorithm summary	How it determines number of clusters (*k*)	Advantages	Disadvantages	Representative studies
Hierarchical Clustering	An unsupervised method for grouping similar data points by building a tree-like structure called a dendrogram. It works either bottom-up (agglomerative), where each point starts as its own cluster and the closest clusters are repeatedly merged, or top-down (divisive), where all points start in one cluster that is repeatedly split. The algorithm relies on a distance metric (such as Euclidean distance) to measure similarity and a linkage method (such as single, complete, average, or Ward’s) to decide how distances between clusters are computed.	Hierarchical clustering does not require specifying *k* in advance. The dendrogram provides a full hierarchy of clusters at different similarity levels, and k is chosen by placing a cutoff (threshold) on this tree, either visually (e.g., by identifying large jumps in merge distance) or using quantitative criteria such as the silhouette score, inconsistency coefficient, or a predefined distance threshold.	• Does not require the number of clusters to be specified in advance • Produces a dendrogram that reveals hierarchical relationships among data points • Flexible: different linkage criteria capture different notions of similarity • Deterministic in the agglomerative case (no random initialization) • Naturally uncovers multilevel, nested cluster structures • Can handle non-spherical / irregularly shaped clusters (unlike k-means, which prefers roughly spherical clusters) • Well suited for exploratory analysis on small to medium datasets, where the dendrogram is easy to inspect	• Computationally expensive for large datasets • Sensitive to noise and outliers, particularly in high-dimensional spaces • Once merged/split, clusters cannot be undone (greedy process) • Results depend heavily on the choice of linkage and distance metrics • Distance measures become less meaningful in high-dimensional spaces; dimensionality reduction is often required.	Uribe et al., 2016, 2018; Guo et al., 2020; Wang L. et al., 2020; Inguanzo et al., 2021; Cao et al., 2022; Inguanzo et al., 2024; Pan et al., 2024; Zhu et al., 2025; Vijayakumari et al., 2026
SuStaIn (Subtype and Stage Inference) (Young et al., 2018)	An unsupervised model that jointly performs clustering and disease progression modelling. It assumes that the cohort is composed of several subtypes, each following its own ordered trajectory of biomarker changes over disease stages. For each subtype, SuStaIn models biomarker evolution using piecewise-linear z-score trajectories over an arbitrary disease timescale. Subtype-specific event sequences and associated parameters are estimated using likelihood-based optimisation, including an expectation-maximization (E-M) step to refine the ordering of biomarker events, while Markov Chain Monte Carlo (MCMC) sampling is used to quantify uncertainty. The algorithm iteratively assigns individuals probabilistically to subtypes and stages, refines subtype-specific progression patterns, and returns both the subtype trajectories and, for each subject, posterior probabilities of subtype and stage.	SuStaIn does not fix the number of subtypes *a priori*. Instead, models with different numbers of subtypes are fit hierarchically (e.g., starting from one subtype and incrementally splitting clusters), and ten-fold cross-validation is used to evaluate out-of-sample likelihood-based information criteria. *k* is chosen as the model that provides the best cross-validated fit to the data while avoiding overfitting, effectively selecting the maximum number of subtypes that the data can support.	• Simultaneously captures phenotypic (subtype) and temporal (stage) heterogeneity in a single framework • Can infer detailed progression patterns from cross-sectional data, without requiring longitudinal follow-up • Provides probabilistic subtype and stage assignments, together with uncertainty estimates (via MCMC) • Reveals biologically meaningful subtypes that can align with genotype or neuropathological patterns, and can uncover within-group heterogeneity • Improves prediction of clinical outcomes compared with subtypes-only or stages-only models • Applicable to any set of scalar biomarkers (e.g., regional MRI volumes), making it flexible across diseases	• Assumes independent biomarker variance, which may not fully reflect biologically correlated processes (though simulations suggest some robustness) • Uses an arbitrary relative timescale; absolute disease time cannot be inferred from cross-sectional data alone • Assumes a finite set of discrete progression patterns; when the true biology is more continuous, the inferred subtypes only approximate that variability • Computationally intensive due to hierarchical model fitting, EM optimization, and MCMC-based uncertainty estimation • Requires relatively large, high-quality datasets for stable estimation of subtype trajectories	Sakato et al., 2024; Park et al., 2025; Shawa et al., 2025
HYDRA (Heterogeneity through Discriminative Analysis) (Varol et al., 2017)	A semi-supervised max-margin framework that simultaneously performs binary classification (patients vs controls) and subtype discovery. HYDRA replaces a single separating hyperplane with a convex polytope formed by multiple linear Support Vector Machines-like hyperplanes. Each hyperplane (face of the polytope) corresponds to a candidate disease subtype: pathological samples are assigned to the hyperplane they lie closest to in margin space. This allows HYDRA to disentangle heterogeneity by capturing distinct, discriminative patterns of abnormality rather than clustering based on global anatomical similarity.	HYDRA does not predefine the number of subtypes. Instead, models are fit for a range of *k* values, and cluster stability is used for model selection. Stability is quantified via cross-validated Adjusted Rand Index (ARI) across repeated runs with different initializations and folds. The value of k that yields the most stable clustering (reflecting reproducible, intrinsic structure in the data) is selected as the optimal number of subtypes.	• Integrates supervision and clustering in a unified framework • Captures disease heterogeneity by modeling multiple discriminative boundaries • Handles nonlinear separability by composing multiple linear hyperplanes into a convex polytope classifier. • Provides reproducible subtypes validated across structural MRI and genetic data. • Applicable to high-dimensional datasets (e.g., neuroimaging and genetic data) • Less susceptible to covariate-driven variation than unsupervised clustering, because subtypes are defined by discriminative patterns between patients and controls.	• Computationally more intensive due to iterative max-margin optimization and repeated initializations. • Requires labeled data for the primary classification (e.g., patients vs. controls). • Optimal K still requires model selection and stability validation • Performance may degrade with small sample sizes or high noise because subtype boundaries rely on stable discriminative patterns. • Sensitive to confounds (e.g., age, sex) if not corrected beforehand, since the model itself does not adjust for covariates.	Vijayakumari et al., 2025b
K-Means Clustering	An unsupervised algorithm that partitions data into *k* clusters by minimizing within-cluster variance. It operates iteratively by initializing *k* centroids (typically randomly), assigning each data point to the nearest centroid, updating the centroids as the mean of their assigned points, and repeating this process until the centroids stabilize or changes in within-cluster variance become minimal.	K-means requires *k* to be specified in advance. The choice of *k* is typically guided by model selection heuristics such as the elbow method (examining the rate of decrease in within-cluster sum of squares), the silhouette coefficient, the gap statistic, or stability-based measures across multiple runs. These metrics help identify the value of *k* that balances model simplicity and cluster separation.	• Simple, fast, and computationally efficient • Scales well to large datasets • Works well when clusters are compact, spherical, and similarly sized • Converges quickly due to its iterative refinement framework • Interpretable cluster centroids (means)	• Requires specifying *k* in advance • Sensitive to initialization; poor starting centroids can lead to suboptimal clustering • Performs poorly on non-spherical, overlapping, or imbalanced clusters • Struggles with outliers and noise • Distance metrics become less meaningful in high-dimensional space, so dimensionality reduction is often required	Vijayakumari et al., 2025a

ARI, Adjusted Rand Index; EM, expectation-maximization; HYDRA, heterogeneity through discriminative analysis; k, number of clusters; MCMC, Markov Chain Monte Carlo; MRI, magnetic resonance imaging; PD, Parkinson’s disease; SuStaIn, Subtype and Stage Inference.

## 4 Methodological considerations

Translating this conceptual model into a practical pipeline requires several methodological choices. The choice of ML methodology can profoundly influence subtyping outcomes. Key decisions include: (1) feature definition/selection and dimensionality reduction strategies, which determine what aspects of brain characteristics are prioritized; (2) the clustering algorithm itself (e.g., hierarchical or k-means), each with distinct assumptions about subtype structure; (3) how the number of subtypes is determined, with metrics such as silhouette scores or gap statistics often yielding different solutions; (4) sample and acquisition characteristics, including disease stage, medication status, scanner/site effects with harmonization, intracranial volume adjustment for morphometry, atlas/parcellation, and cohort size; and (5) validation approaches, ranging from internal stability and resampling-based assessments to external replication in independent cohorts.

The following sections examine how these methods have been applied to specific MRI modalities and what insights have emerged regarding PD heterogeneity. Table 2 summarizes the details of MRI-based subtyping studies.

**TABLE 2:** Summary of MRI-based subtyping studies in Parkinson’s disease.

Study references	PD cohort description	Clustering method	Imaging features	PD sample (n)	Number of features	Key findings
Uribe et al., 2016	Single-center, nondemented, mild-moderate	Hierarchical clustering	Cortical thickness	88	327,684	Three subtypes of cortical atrophy in non-demented PD: (1) parieto-temporal thinning with worse cognitive performance and more depression; (2) occipital-frontal atrophy linked to younger onset; (3) no cortical atrophy.
Uribe et al., 2018	*de novo*, drug-naïve PPMI cohort	Hierarchical clustering	Cortical thickness	77	360	Two cortical atrophy patterns in early PD: (1) anterior thinning (orbitofrontal, anterior cingulate, temporal) with normal cognition; (2) posterior thinning (parietal, occipital, postcentral) associated with poorer cognition and greater motor symptoms
Uribe et al., 2019	Longitudinal follow-up of their 2016 study	Hierarchical clustering	Cortical thickness	45	NA	Pattern 1 (parieto-temporal) had greater attrition and worse functional and cognitive decline; Pattern 2 (frontal–occipital, younger onset) showed limited cortical thinning; Pattern 3 (initially non-atrophic) demonstrated extensive temporo-parietal thinning.
Wang L. et al., 2020	A *de novo*, drug-naïve at baseline, longitudinally followed PPMI cohort.	Hierarchical clustering	DBM	314	79	Two neuroanatomic biotypes in early PD: (1) biotype 1 showed smaller subcortical volumes, more severe motor and nonmotor impairment, greater autonomic dysfunction, worse RBD, and faster disease progression across motor, cognitive, and dopaminergic imaging measures; (2) biotype 2 showed larger subcortical volumes, milder baseline symptoms, and slower disease progression rate.
Guo et al., 2020	Single-center, mixed-stage	Hierarchical clustering	Whole-brain fiber connectivity (FN × mean FA)	134	2 CCA components (from 5995 features)	Three PD subtypes: (1) mild, (2) severe motor-dominant, and (3) severe depression-dominant. The depression-dominant subtype showed widespread disruptions in functional and structural connectivity.
Inguanzo et al., 2021	Single-center, nondemented, mild–moderate	Hierarchical clustering	GMV + FA	62	85	Three PD subtypes: (1) widespread cortical and subcortical gray-matter atrophy with white matter FA reductions and marked cognitive impairment; (2) frontal and temporal cortical atrophy associated with more selective neuropsychological deficits; and (3) no detectable structural atrophy or cognitive impairment.
Cao et al., 2022	Single-center, mild–moderate	Hierarchical clustering	ALFF + GMV	86	34	Two PD subtypes: (1) diffuse malignant subtype characterized by reduced ALFF in visual cortex and widespread GMV reduction with severe motor and cognitive impairment; (2) mild subtype showed increased ALFF in frontal, temporal, sensorimotor cortices with slight GMV loss and mild motor and cognitive impairment.
Inguanzo et al., 2024	Multi-cohort PPMI: *de novo*, unmedicated Barcelona UB-Clínic: medicated, advanced KIMOVE: mixed stage and mixed medication status EXPANd: mild–moderate	Hierarchical clustering	Cortical thickness + subcortical GMV	633	41	Unadjusted analysis (without controlling for global atrophy) identified three PD subtypes reflecting increasing degree of atrophy, associated with increasing age and motor impairment. Adjusted analysis (controlling for global atrophy) revealed eight distinct MRI-based subtypes characterized by regional atrophy patterns. Subtypes with basal ganglia or parieto-occipital atrophy showed faster motor progression, while the parieto-occipital subtype also showed cognitive decline.
Pan et al., 2024	*de novo*, drug-naïve PD with ≥3-year MRI follow-up (PPMI)	Hierarchical clustering	Rate of regional GMV loss	107	254	Two subtypes: (1) moderate atrophy in the prefrontal and temporal lobes; (2) more widespread and faster atrophy, particularly in the temporal and subcortical regions, with increased motor and non-motor symptom progression.
Sakato et al., 2024	Multi-cohort KUHP: university & NCNP hospital: early to long-duration PD PPMI: *de novo;* drug-naïve	SuStaIn	GMV	504	45	Three subtypes: (1) Neocortical, (2) Limbic, and (3) Brainstem. The neocortical subtype showed early frontal–parietal cortical atrophy that spread across the neocortex, relatively sparing striatum, pallidum, accumbens, and brainstem, and it was associated with older onset and cognitive decline. The limbic subtype showed early involvement of amygdala, accumbens, striatum, and temporal cortex, later extending to parietal and frontal regions, and this group also exhibited cognitive deficits. The brainstem subtype showed a caudal-to-rostral pattern starting in the brainstem and progressing to limbic and then cortical areas, and it was characterized by a higher autonomic symptom burden, with relatively milder cognitive involvement. Subtype assignments were largely stable at 2- and 4-year follow-up.
Vijayakumari et al., 2025b	*de novo*, drug-naïve PPMI cohort	HYDRA	QA (brainstem)	124	14	Two subtypes: (1) Higher QA (preserved axonal integrity) and lower RBD severity; (2) Reduced QA (axonal degeneration) across all tracts and more severe RBD.
Park et al., 2025	Single-center, relatively short PD duration	SuStaIn	Cortical thickness + subcortical GMV	565	16	Three subtypes: (1) cortex-first, demonstrating cortical thinning followed by atrophy in subcortical regions, with faster disease progression and higher prevalence of RBD and dyskinesia; (2) deep grey-first, showing subcortical atrophy first, followed by cortical thinning; (3) no significant atrophy progression.
Shawa et al., 2025	Multi-cohort framework ENIGMA-PD: multi-site; mixed-stage PD PPMI: *de novo*; untreated early PD; longitudinal	SuStaIn	Cortical thickness + subcortical GMV	1100 (training, ENIGMA-PD) and 584 (testing, PPMI)	14	Four subtypes: (1) subcortical subtype, with earliest atrophy in caudate, pallidum, and putamen; (2) limbic subtype, with early ventricular/hippocampal/amygdala involvement spreading to association cortices; (3) cortical subtype, with predominant cortical/lobar atrophy; and (4) sub-threshold/no-atrophy group. Clinical scores (motor, cognitive, autonomic, RBD) were generally worse in any of the three atrophy subtypes than in the sub-threshold group, but showed little separation among the three atrophy patterns, suggesting overall atrophy burden was more clinically relevant than spatial pattern. These four groups were reproduced in the independent PPMI cohort (*n* = 584), and subtype assignments were largely stable longitudinally (∼84%).
Vijayakumari et al., 2025a	A *de novo*, drug-naïve at baseline, longitudinally followed PPMI cohort.	K-means clustering	Multivariate GMV distances	114	6	Two subtypes: (1) higher GMV atrophy in frontal and subcortical regions, with poorer cognition and faster progression of postural instability and gait disturbance; (2) minimal atrophy and no clinical deterioration over follow-up.
Zhu et al., 2025	Single-center, early-stage	Hierarchical clustering	Cortical thickness + FA + QSM (subcortical iron content)	179	126	Two subtypes: (1) early-deterioration with frontotemporal atrophy, parietal thickening, reduced FA, and increased subcortical iron, with worse baseline motor/non-motor scores and a trend toward faster memory decline; (2) early-compensatory with rostral middle frontal atrophy, parieto-occipital thickening, increased FA, and normal subcortical iron content, with mild baseline symptoms but faster motor and autonomic symptom progression on follow-up; findings were reproduced in an independent cohort.
Vijayakumari et al., 2026	A *de novo*, drug-naïve at baseline, longitudinally followed PPMI cohort.	Hierarchical clustering	ISO (subcortical motor regions)	156	12	Two subtypes: (1) lower baseline ISO across subcortical motor regions; (2) higher baseline ISO with greater baseline rigidity and bradykinesia. No significant subtype differences were observed in longitudinal change in ISO or motor progression over 4 years.

ALFF, amplitude of low-frequency fluctuation; Barcelona UB-Clínic, Cohort from Hospital Clínic, Barcelona; CCA, Canonical Correlation Analysis; DBM, deformation-based morphometry; ENIGMA, Enhancing NeuroImaging Genetics through Meta-Analysis; EXPANd, cohort from the Exercise in Parkinson’s disease and Neuroplasticity trial; FA, fractional anisotropy; FN, fiber number; GMV, gray matter volume; HYDRA, Heterogeneity through Discriminative Analysis; ISO, isotropic diffusion component; KIMOVE, cohort from the Karolinska Imaging in Movement Disorders study; KUHP, Kyoto University Hospital; NCNP, National Center of Neurology and Psychiatry; PD, Parkinson’s disease; PPMI, Parkinson’s Progression Markers Initiative; QA, quantitative anisotropy; QSM, quantitative susceptibility mapping; RBD, REM (rapid eye movement) sleep behavior disorder; SuStaIn, Subtype and Stage Inference; WM, white matter.

### 4.1 Structural (T1-weighted) MRI–based subtyping

Early MRI-based subtyping efforts in PD focused on capturing structural heterogeneity in non-demented patients using cortical thickness measures (Uribe et al., 2016, 2019). A seminal study by Uribe et al. identified three anatomically distinct subtypes characterized by parieto-temporal, frontal-occipital, or minimal cortical atrophy (Uribe et al., 2016). The parieto-temporal subtype showed markedly poorer cognitive performance, especially in semantic fluency, whereas the frontal-occipital subtype was linked to younger age at onset. Longitudinal follow-up confirmed the prognostic relevance of these patterns (Uribe et al., 2019). The minimal-atrophy group exhibited gradual and widespread cortical thinning, the frontal-occipital group showed more localized progression, and the parieto-temporal group experienced the fastest cognitive and functional decline, with many patients progressing to dementia. Subsequent work by the same group extended this framework to early, untreated PD, revealing two primary subtypes: one with anterior cortical thinning and relatively preserved cognition, and another with posterior thinning linked to cognitive impairment and more severe motor features (Uribe et al., 2018). Together, these studies established that structural brain changes in PD follow distinct neurodegenerative and clinical trajectories.

A series of subsequent studies further refined our understanding of structural heterogeneity in PD. One study identified two subtypes based on the extent of subcortical atrophy: one with widespread volume loss linked to greater motor, cognitive, and autonomic burden, and another with relatively preserved subcortical structure and slower clinical decline. These findings suggest that subcortical “reserve” may buffer against neurodegeneration in early PD (Wang L. et al., 2020). A multi-cohort study encompassing different disease stages and clinical contexts (including *de novo*/unmedicated and medicated patients) used cortical thickness and subcortical volume measures to derive MRI-based PD subtypes. In this study, global atrophy, representing the overall burden of structural brain loss, was used as a proxy for neurodegenerative severity. Regional cortical thickness measures were adjusted for each individual’s mean cortical thickness, while regional gray-matter volumes were adjusted for total gray-matter volume after accounting for intracranial volume. Without this adjustment, clustering identified three reproducible subtypes along a gradient from minimal to widespread atrophy, with the most atrophied subtype comprising older patients with more severe motor symptoms. After adjustment, eight anatomically specific subtypes emerged. In particular, subcortical-predominant and parieto-occipital profiles were linked to faster motor decline, with the latter also showing cognitive worsening. Although some age differences remained and the regional subtypes were less stable across cohorts, these findings demonstrate that adjusting for global atrophy can reduce the influence of overall disease severity and improve the detection of region-specific patterns, thereby affecting the number, anatomical specificity, and reproducibility of MRI-derived subtypes (Inguanzo et al., 2024).

Another study applied a patient-specific multivariate gray-matter volumetric (M_GMV_) approach to derive low-dimensional, region-specific atrophy indices. Mahalanobis-distance scores (a statistical measure quantifying how far each patient’s regional volume deviates from healthy controls across multiple brain regions simultaneously) were computed for six regions (frontal, subcortical, parietal, medial-temporal, lateral-temporal, and occipital), reducing high-dimensional region-of-interest (ROI) data into a compact, interpretable feature set. Higher scores reflected greater regional atrophy. Clustering these features revealed two early-stage subtypes: one with frontal and subcortical atrophy, associated with poorer global cognition and faster PIGD progression over 48 months, and another with preserved structure and slower progression. These findings suggest that dimensionality-reduced, region-specific atrophy profiles can provide a robust and clinically meaningful framework for PD subtyping (Vijayakumari et al., 2025a).

These subtyping studies have mostly relied on cross-sectional T1-weighted MRI to define anatomically meaningful PD subgroups and then used available longitudinal clinical (and, less often, imaging (Uribe et al., 2019)) follow-up data to test whether those MRI-defined subtypes diverge over time. Extending subtyping into the temporal domain, Pan et al. estimated patient-specific regional gray-matter atrophy rates from baseline and ≥3 years (mean ∼4 years) of longitudinal MRI and applied clustering to these rates. They identified two progression phenotypes: (1) a moderate, fronto-temporal–predominant trajectory, and (2) a more widespread, faster trajectory with greater lateral temporal, hippocampal, and thalamic involvement. The widespread/faster subtype showed more rapid worsening of motor and non-motor features, including autonomic dysfunction, depressive symptoms, and memory, over follow-up. Their findings suggested that MRI-based progression patterns can help stratify PD for more personalized monitoring and treatment (Pan et al., 2024).

Complementing this longitudinal study, several groups have used cross-sectional T1-weighted MRI data with progression modeling (the SuStaIn algorithm (Young et al., 2018)) to reconstruct latent subtypes and infer the sequence of regional involvement for each subtype. Unlike traditional cross-sectional clustering, which identifies patient groups based on their current imaging profile, disease-progression modeling reconstructs the temporal sequence in which brain regions become affected within each subtype. This approach leverages the variability across patients at different disease stages to estimate a common underlying progression trajectory, essentially transforming cross-sectional data into pseudo-longitudinal staging information. One study identified three spatiotemporal atrophy subtypes: neocortical (early frontal/parietal atrophy), limbic (early amygdala/accumbens/striatum/temporal atrophy), and brainstem (caudal to rostral spread). Longitudinal follow-up (2–4 years) showed that most patients remained in the same subtype and advanced in stage, suggesting that the cross-sectional subtypes reflected true progression. Patients in the neocortical subtype were generally older and more cognitively susceptible, and the three patterns exhibited similarities to Lewy-body distributions, thereby affirming the biological validity of these subtypes (Sakato et al., 2024). Another study found two distinct progression trajectories: a cortex-first subtype with early cortical thinning, faster progression, and a higher prevalence of RBD and levodopa-induced dyskinesia, and a deep-grey–first subtype with earlier subcortical/amygdala changes and slower progression. In both subtypes, the sensorimotor and auditory cortices were the earliest cortical regions to change, the amygdala was the earliest subcortical site, and about one-third of patients showed little or no MRI-visible atrophy, forming a low-atrophy group (Park et al., 2025). Although these results are encouraging, the authors noted that longitudinal studies in prospective cohorts will be needed to confirm subtype stability and staging. Finally, Shawa et al. leveraged two of the largest PD MRI datasets (ENIGMA-PD for training and PPMI for longitudinal testing) and identified three distinct atrophy subtypes (subcortical, limbic, and cortical), along with a substantial no-atrophy group. Patients with visible atrophy tended to show worse clinical progression than those without, although differences among the three atrophic patterns were modest. The authors concluded that T1-weighted MRI alone captures a real but relatively coarse PD heterogeneity signal and that more fine-grained phenotyping will require multimodal imaging and/or molecular biomarkers (Shawa et al., 2025).

Taken together, a set of recurring themes emerges. First, most analyses identify 2 to 3 structural subtypes, with a reproducible distinction between patients showing minimal atrophy and those with more pronounced regional vulnerability. Second, posterior-predominant (parieto-temporal, limbic) patterns consistently associate with cognitive impairment and faster decline, while frontal and subcortical involvement relates more strongly to motor progression (Uribe et al., 2016, 2018; Wang L. et al., 2020; Vijayakumari et al., 2025a). Third, a substantial proportion of early-stage PD patients show minimal detectable atrophy on T1-weighted imaging, suggesting either genuine structural preservation or that conventional structural MRI lacks sensitivity to capture their pathology. Finally, factoring out global atrophy reveals more anatomically specific subtypes, but these are less stable across cohorts, underscoring a tension between biological granularity and practical generalizability (Inguanzo et al., 2024).

In this context, T1-based structural subtypes appear to be a plausible starting point for cohort stratification or hypothesis-driven trial enrichment, for example, by oversampling posterior/neocortical cases for cognitive endpoints or frontal–subcortical cases for motor outcomes.

### 4.2 Diffusion MRI-based subtyping

Diffusion MRI can capture microstructural integrity *in vivo* and is sensitive to subtle changes that may not be visible on conventional T1-weighted imaging (Alexander et al., 2001, 2007; Pasternak et al., 2009; Yeh et al., 2010, 2013; Jeurissen et al., 2019). To date, only a few groups have used diffusion-derived measures such as tractography-derived streamline count (a tractography-based estimate of white matter connection strength), fractional anisotropy (FA; an index of white matter integrity, with lower values suggesting axonal or myelin disruption), quantitative anisotropy (QA; an alternative index of axonal integrity), and isotropic diffusion (ISO; a diffusion-derived measure of direction-independent isotropic water diffusion that is sensitive to microstructural alterations) to derive biologically meaningful PD subtypes.

Using DTI-derived whole-brain fiber connectivity, Guo et al. calculated edge weights by multiplying the tractography-derived streamline count, termed fiber number (FN) in the original study, by the mean FA along the reconstructed interregional pathways. They first identified clinically relevant motor- and depression-related connectivity patterns and then obtained three clinically separable subgroups: a relatively mild group, a motor-dominant group, and a depression-dominant group (Guo et al., 2020). The depression-dominant subtype showed the most pronounced white-matter microstructural abnormality. When resting-state fMRI was examined *post hoc*, more widespread functional dysconnectivity was also observed, suggesting that diffusion-defined disruptions map onto network-level dysfunction. However, these findings should be interpreted cautiously because tractography-derived streamline counts are indirect estimates of white-matter connectivity rather than direct measures of axonal fibers and can be influenced by methodological factors related to tract reconstruction and tracking algorithms (Schilling et al., 2018, 2020).

Using QA-based tractography in the brainstem, one study identified two PD subtypes: one with relatively preserved QA and one with widespread QA reductions, the latter showing a higher RBD burden, consistent with greater involvement of REM-regulatory pathways (Vijayakumari et al., 2025b). In a separate analysis, the same group applied clustering to ISO values from subcortical motor regions and again identified two subtypes, a “low-ISO” and a “high-ISO” group, with the high-ISO group demonstrating more severe baseline rigidity and bradykinesia. However, longitudinal analyses over 4 years, conducted using baseline-adjusted change-score models that accounted for initial motor severity, baseline ISO values, age, and sex, did not reveal significant differences between subtypes in subsequent motor progression or imaging change (Vijayakumari et al., 2026).

Given the limited evidence base, with only three studies meeting our inclusion criteria, and the heterogeneity in tractography metrics, including fiber connectivity, QA, and ISO, as well as differences in anatomical targets, no stable diffusion MRI-derived subtyping framework in PD can currently be reliably identified. These findings should therefore be regarded as preliminary.

### 4.3 Multimodal MRI-based subtyping

Multimodal imaging approaches are emerging as a promising tool for subtyping because they offer a more integrative framework for characterizing neurodegeneration that spans gray matter morphology, white matter integrity, and functional activity measures. By combining modalities, these methods can help overcome the limitations of single-modality analyses and capture complementary biological dimensions of disease heterogeneity.

In a multimodal analysis, cortical and subcortical gray matter volumes were combined with white matter FA to identify three subtypes, with gray matter differences being the dominant separator: (1) a diffuse atrophy group with widespread cortical and subcortical volume loss, accompanied by relatively modest white matter integrity reductions, and pronounced cognitive impairment; (2) a frontal-temporal group with atrophy mainly confined to frontal and temporal cortices and a more circumscribed pattern of cognitive deficits; and (3) a “resilient” group without detectable imaging abnormalities and with preserved cognition (Inguanzo et al., 2021). In another multimodal analysis, amplitude of low-frequency fluctuation (ALFF; a measure of spontaneous neural activity) from resting-state fMRI was combined with gray matter volume from structural MRI. This approach identified a “diffuse malignant” subtype marked by widespread gray matter volume reduction and reduced ALFF in visual cortices, and a “mild” subtype showing increased ALFF in frontal, temporal, and sensorimotor regions and only slight gray matter volume loss. The malignant subtype showed worse motor and cognitive scores, indicating that multimodal MRI captures clinically relevant disease heterogeneity (Cao et al., 2022).

More recently, a trimodal approach combining cortical thickness, diffusion metrics, and subcortical iron deposition from quantitative susceptibility mapping (QSM) identified two early-stage subtypes. (1) An ‘early-deterioration’ group with frontotemporal atrophy and parietal thickening, diffuse FA reductions, and elevated iron accumulation. This subtype was associated with more severe baseline symptoms and a trend toward faster memory decline. (2) An ‘early-compensatory’ group with preserved iron, selective frontal atrophy and parieto-occipital thickening, and increased FA in specific tracts, showing initially milder symptoms but later acceleration of motor and autonomic decline (Zhu et al., 2025).

In summary, multimodal MRI studies in PD provide additional insight compared with single-modality analyses by capturing complementary aspects of neurodegeneration (e.g., cortical atrophy, white-matter changes, and iron accumulation). However, the limited number of studies and heterogeneous feature sets, despite all using hierarchical clustering, preclude firm conclusions about the specific contribution of multimodal imaging.

### 4.4 Critical synthesis and clinical interpretation

The studies reviewed here provide distinct perspectives on PD heterogeneity, although the strength of evidence varies considerably across MRI modalities. T1-weighted MRI currently provides the most mature and clinically interpretable evidence base. Multiple independent studies have consistently identified subgroups characterized by minimal detectable atrophy, posterior cortical involvement, frontal-subcortical changes, or more widespread neurodegeneration. Importantly, these structural patterns show associations with clinically relevant outcomes, particularly cognitive impairment and motor progression (Uribe et al., 2016, 2019; Inguanzo et al., 2024; Vijayakumari et al., 2025a). Although T1-weighted MRI is easy to acquire and process and is highly effective for quantifying macrostructural features (cortical thickness, regional gray matter volume, and subcortical volume), it cannot directly assess tissue microstructure or functional network alterations.

Diffusion MRI has the potential to address some of these limitations by detecting microstructural abnormalities that are not apparent on conventional structural MRI. However, the evidence is currently fragmented and difficult to integrate. The three eligible studies identified subtypes using fundamentally different diffusion MRI features, including connectivity-based, anisotropy-based, and free-water-related metrics (Guo et al., 2020; Vijayakumari et al., 2025b; Vijayakumari et al., 2026). These features capture different aspects of tissue organization and pathology, making direct comparisons across studies difficult. Moreover, the field lacks a common framework regarding which diffusion metrics, preprocessing strategies, and anatomical targets should be prioritized for subtyping. Future work should determine whether alternative diffusion metrics, including mean diffusivity, axial diffusivity, and radial diffusivity, or more advanced multicompartment models such as neurite orientation dispersion and density imaging, provide more reproducible or clinically informative subtype solutions.

Resting-state functional MRI represents a notable gap in the current literature. Despite growing recognition that PD involves altered functional network connectivity (Baggio et al., 2015; Akgüller et al., 2024; Bin et al., 2026), no eligible study used resting-state connectivity measures as the primary modality for subtype discovery. Instead, functional MRI was incorporated into multimodal analyses or used to further characterize subtypes identified from structural MRI data (Guo et al., 2020; Cao et al., 2022). Resting-state fMRI may capture disease-related alterations in functional network organization, such as patterns of network disruption and potential compensatory reorganization, that are not reflected by structural measures alone (Hohenfeld et al., 2018). At present, its contribution to PD subtyping remains largely unexplored. Future studies should determine whether functional connectivity-based subtypes provide prognostic or mechanistic insights beyond those provided by structural MRI.

Multimodal MRI is conceptually attractive because it integrates complementary biological information regarding brain morphology, white matter integrity, iron deposition, and functional organization. From a biological perspective, such approaches may be better aligned with the multisystem nature of PD than any single modality (Cilia et al., 2026). However, the available evidence remains limited. Existing studies use different combinations of imaging modalities, feature-selection strategies, and clustering methods, making direct comparisons difficult (Cao et al., 2022; Zhu et al., 2025). More importantly, it remains unclear whether multimodal approaches improve subtype reproducibility, longitudinal prediction, or clinical utility compared with simpler single-modality models. The increased acquisition burden, cost, and analytical complexity associated with multimodal frameworks are not yet supported by clear evidence of added clinical value. Future studies should directly compare single-modality and multimodal approaches within the same cohorts to quantify the incremental contribution of each imaging technique and determine whether multimodal models provide meaningful advantages over more parsimonious alternatives.

Differences across studies should also be interpreted in the context of the analytical framework employed. Hierarchical clustering and k-means identify groups of patients with similar imaging characteristics, whereas HYDRA identifies distinct patterns of patient-control deviation, and SuStaIn simultaneously models disease subtype and disease stage. As these methods are designed to capture different aspects of disease heterogeneity and rely on different underlying assumptions, they should not be expected to yield identical subtype solutions. Consequently, inconsistencies across studies do not necessarily reflect methodological shortcomings. Instead, they may reflect differences in the aspects of disease heterogeneity captured by each algorithm, including disease severity, anatomical distribution of abnormalities, temporal progression, and deviation from healthy control populations.

The clinical interpretation of MRI-derived subtypes requires caution. A minimal-atrophy subtype may reflect relatively early disease, compensatory mechanisms, or the limited sensitivity of current imaging methods. Conversely, widespread atrophy may indicate a greater neurodegenerative burden or a more advanced disease stage rather than a distinct pathophysiological form of PD. Similar interpretive challenges apply to diffusion- and connectivity-based subtypes, where abnormalities may reflect disease severity, compensatory reorganization, or modality-specific measurement effects. Therefore, MRI-derived subtypes should not yet be assumed to represent biologically distinct subgroups of PD. Moreover, none of the proposed MRI-based subtype frameworks has been sufficiently validated for diagnosis, individualized prognosis, or treatment selection, and external validation across independent cohorts remains limited. Currently, their primary value lies in demonstrating that the clinical heterogeneity of PD is associated with measurable variability in neuroanatomical and network-level abnormalities.

## 5 Challenges and limitations in MRI-based subtyping

While MRI-based subtyping has provided important insights into the biological heterogeneity of PD, substantial methodological challenges continue to hinder the development of a stable and clinically useful subtype framework. In this section, we critically examine the key technical, statistical, and conceptual barriers that currently limit the reproducibility and generalizability of MRI-derived subtypes.

### 5.1 Data collection burden in PD

Unlike conditions such as multiple sclerosis, stroke, or brain tumors, where MRI is central to diagnosis and monitoring, PD is primarily diagnosed through clinical assessment of motor symptoms and response to dopaminergic therapy. MRI is not required in PD diagnostic criteria (Postuma et al., 2015) and, when used in clinical care, typically employed to rule out vascular or atypical parkinsonian syndromes rather than to characterize PD itself (Chougar et al., 2020; Tolosa et al., 2021). As a result, routine scans often use lower-resolution sequences with limited anatomical coverage and variable, site-specific acquisition parameters. This leads to heterogeneity in slice thickness, contrast weighting, and reconstruction methods, making data pooling and harmonization difficult. Moreover, clinical protocols are optimized for qualitative interpretation and typically lack the standardization, resolution, and quantitative sequences that may provide complementary information for subtyping. These include QSM, diffusion MRI, and resting-state functional MRI. We highlight resting-state rather than task-based fMRI because it does not require patients to reliably perform a motor or cognitive task, making it more feasible for application across PD cohorts with varying disease severity. Neuromelanin-sensitive MRI is also an attractive option because it provides a PD-relevant measure of neuromelanin-containing nuclei, particularly the substantia nigra pars compacta and locus coeruleus (Hwang et al., 2023), but has not yet been applied in data-driven subtyping studies. Acquiring these dedicated quantitative sequences may require extended scan times, protocol development, ethical approvals, and participant recruitment, all of which add logistical and financial burdens (Politis, 2014; Heim et al., 2017). Beyond protocol constraints, PD-related tremor and dyskinesia frequently increase motion artifacts, particularly in longer or high-resolution sequences. Together, these factors hinder the collection of large, high-quality imaging datasets needed to develop robust MRI-based subtypes in PD.

### 5.2 Sample size limitations and the curse of dimensionality

Acquiring high-quality MRI data in PD is resource-intensive, often limiting sample sizes, especially in single-site studies. This becomes a major challenge when paired with the high dimensionality of neuroimaging features. Subtyping studies commonly involve tens of thousands of cortical vertices, hundreds of ROI metrics, or complex connectivity matrices. As shown in Table 2, many PD studies include far more imaging features than participants (Uribe et al., 2018, 2019; Inguanzo et al., 2021; Pan et al., 2024), creating an imbalance that undermines cluster stability and reproducibility. Some studies have addressed this by limiting the number of regions analyzed and/or using dimensionality reduction (Wang Y. et al., 2020; Zhu et al., 2025) or multivariate approaches (Vijayakumari et al., 2025a) to generate more compact feature sets. When dimensionality remains high, it exacerbates the curse of dimensionality, meaning clustering in sparse feature spaces becomes noise-sensitive and prone to instability (Bouveyron et al., 2007). As a result, models may overfit noise, subtypes may shift with minor analytic changes, and replication may fail, limiting clinical translation. Although techniques like principal component analysis or region averaging can reduce dimensionality, they are not always sufficient (Jolliffe and Cadima, 2016; Salem and Hussein, 2019). For instance, compressing 10,000 features (e.g., brain vertices) into 500 components still yields a high feature-to-sample ratio in studies with only 100-150 subjects. Addressing this challenge requires either larger, harmonized multi-site datasets or methods built to handle high-dimensional, low-sample-size data (Shen et al., 2016).

### 5.3 Scanner effects and the need for data harmonization

A major gap in MRI-based subtyping studies is the inconsistent use of harmonization techniques to control scanner- and site-related variability. Multi-site datasets (e.g., PPMI, ENIGMA) boost sample size and generalizability but also introduce non-biological variance from differences in hardware, acquisition protocols, and reconstruction algorithms. Several studies (Uribe et al., 2018; Wang L. et al., 2020; Inguanzo et al., 2021; Pan et al., 2024; Shawa et al., 2025) did not report the use of harmonization pipelines, which means scanner effects may have been partially conflated with biological variation. In contrast, recent work (Sakato et al., 2024; Vijayakumari et al., 2025a) has applied tools such as ComBat (Fortin et al., 2017) or NeuroHarmonize (Pomponio et al., 2020) before subtyping, showing that site-related variance can be reduced while preserving biological signal. Without harmonization, clustering algorithms may subtype patients by scanner characteristics rather than the true pathology. Harmonization should therefore be treated as a prerequisite for multi-site subtyping studies, and its omission represents a significant source of bias.

### 5.4 Gaps in MRI subtyping pipelines

Beyond limited sample sizes, inconsistent image-processing methods further complicate MRI-based subtyping. Although most studies include core preprocessing steps – motion correction, spatial normalization, tissue segmentation, and smoothing – their implementation often varies. Key choices, such as software tools, anatomical atlases, and parameter settings, are typically driven by researcher preference. This variability hinders replication and cross-cohort comparisons. Large-scale reproducibility studies show that even small analytic differences can lead to divergent conclusions (Botvinik-Nezer et al., 2020; Schilling et al., 2021), raising concerns about the reliability of MRI-derived subtypes. While innovation is important, the field must address critical questions: Which pipelines are most reproducible across datasets? Where should flexibility end and standardization begin? Clear best practices and transparent workflows are essential for making subtyping clinically meaningful.

### 5.5 Dependence on healthy control reference models

Many current subtyping ML frameworks, particularly those using SuStaIn (Young et al., 2018) or HYDRA (Varol et al., 2017), typically rely on a well-characterized control set to define a normative reference space. A similar dependence arises when studies construct imaging features as Mahalanobis distances from a control group, as in Vijayakumari et al. (2025a). In this setting, the distance is computed using the control mean vector and covariance matrix, so the control sample directly determines what counts as “deviation”. Even when controls are age-matched or demographic effects are regressed out, the shape and quality of the control distribution still determine how “normality” is defined. If the control cohort is small, demographically narrow, or scanned under different conditions, these reference models can impose bias on the resulting subtypes and distort staging precision. In effect, variability in the control group propagates through the entire subtyping pipeline, influencing both the apparent severity and the separability of patient clusters. Given this strong dependence on the control group, future studies should use larger and more representative control cohorts, test how stable their models are when the control sample is changed and consider more advanced normative approaches so that “normality” is not defined by a single small, potentially biased control group.

### 5.6 Arbitrary or heuristic subtype determination

Many clustering algorithms require users to predefine the number of subtypes (*k*), yet there is no universally accepted criterion for choosing *k* in neuroimaging contexts. Heuristic tools, such as the elbow method, silhouette scores, or stability metrics, offer guidance but often yield different solutions (Rousseeuw, 1987; Hennig, 2015). For instance, one dataset might suggest *k* = 2 subtypes by the elbow method but *k* = 4 by silhouette analysis, with no clear theoretical basis for choosing between them. More advanced probabilistic approaches, such as SuStaIn, can select the number of subtypes via model comparison, but they bring their own assumptions and complexities (Young et al., 2018). This uncertainty mirrors a deeper issue: the field has not yet reached consensus on how to best characterize PD heterogeneity (Marras and Lang, 2013).

### 5.7 Overreliance on cross-sectional data and the challenge of temporal stability

Subtyping is intended to identify biologically and clinically meaningful groups that capture distinct disease mechanisms or trajectories. Most studies (Uribe et al., 2018; Inguanzo et al., 2021; Cao et al., 2022; Vijayakumari et al., 2025a,b, 2026) rely on cross-sectional MRI data to infer PD subtypes, assuming that differences observed at a single time point reflect stable, persistent biological categories. PD, however, is a progressive and multifaceted neurodegenerative disorder (Ahlskog, 2024), and this assumption may not hold. In the absence of longitudinal data, it remains unclear whether cross-sectionally defined subtypes persist over time or represent transient phenotypes driven by disease stage, compensatory mechanisms, or treatment effects.

## 6 Future directions

To advance the field of MRI-based subtyping in PD, future studies must continue to prioritize rigorous data quality control, including systematic assessment of motion artifacts, signal-to-noise ratios, and scanner-related variability. Researchers must carefully balance feature-to-sample ratios when working with high-dimensional MRI data and employ robust methods for determining cluster number, including resampling and bootstrapping approaches to ensure stability of subtype assignments (Gana Dresen et al., 2008; Yu et al., 2019). Equally important is the careful control of confounding variables such as age, sex, site effects, and head motion, which is essential to avoid spurious subtypes that reflect technical or demographic differences rather than underlying neurobiological heterogeneity.

Cross-sectional subtyping frameworks must be validated longitudinally to determine whether identified subtypes predict divergent disease trajectories, symptom progression patterns, or differential treatment responses, thus establishing clinical utility. Promising frameworks must undergo external validation in independent cohorts from different populations and scanning protocols, testing stability across disease stages and diverse ethnic populations. Achieving this level of validation requires open science practices with full sharing of preprocessing pipelines, analysis code, and containerized workflows. Only through multi-site replication and community-wide validation can we distinguish robust neurobiological subtypes from site-specific phenomena and build consensus around classifications ready for precision medicine.

Another important phase is to validate whether MRI-defined subtypes correspond to distinct underlying molecular pathology. Future studies should test whether imaging-based subtypes show differential patterns in cerebrospinal fluid or blood markers of α-synuclein, genetic variants, and dopaminergic positron emission tomography imaging, thereby confirming that clusters reflect distinct disease mechanisms. Where available, neuropathological validation can help determine whether specific imaging-defined patterns correspond to distinct distributions of α-synuclein pathology, neuronal loss, or gliosis, thereby strengthening claims that these MRI-derived groups represent biologically meaningful phenotypes (De Pablo-Fernández et al., 2019).

A practical next step is to improve the interpretation and reporting of MRI-derived subtype solutions. Studies should clearly show which anatomical or network-level features distinguish each subtype. The stability and uncertainty of subtype assignments should also be systematically evaluated using methods appropriate to the clustering approach, such as resampling- or bootstrap-based stability analyses (Wen et al., 2025). Probabilistic frameworks can further provide estimates of assignment confidence (Wright et al., 2020). For clinical or trial applications, assignment confidence and reproducibility should be rigorously validated. High-confidence assignments may be more suitable for patient stratification, whereas uncertain cases should be interpreted cautiously.

Bringing MRI subtyping into routine practice will also require simplification. Complex MRI-clustering pipelines need to be distilled into scoring tools or lightweight applications that take a small set of standardized MRI-derived measures and return “Subtype X (Y% probability)” together with a brief clinical interpretation (for instance, higher likelihood of cognitive decline or falls). In principle, such tools could eventually support trial designs by helping to enrich arms with patients whose imaging suggests specific pathophysiological profiles such as more synuclein-driven vs. more inflammatory or network-vulnerability patterns, but this remains an aspirational goal and will require careful prospective validation.

## 7 Conclusions

Data-driven MRI subtyping is beginning to clarify PD heterogeneity, suggesting that the disorder comprises multiple imaging-derived phenotypes with distinct risks and trajectories. The task now is to turn these research-grade findings into a reliable, biologically grounded classification that adds prognostic and therapeutic value beyond current clinical tools. With sustained multidisciplinary work, including standardized MRI pipelines, rigorous longitudinal and external validation, and transparent reporting of subtype stability and assignment uncertainty, MRI subtyping can move from an exploratory tool to a foundation for precision medicine in PD, guiding prognosis and trial design, and ultimately improving outcomes for individual patients.
